# Effects of Dangguibuxue decoction on rat glomerular mesangial cells cultured under high glucose conditions

**DOI:** 10.1186/s12906-017-1774-4

**Published:** 2017-05-25

**Authors:** Xiao-Dan Ren, Ying-Wen Zhang, Xiu-Ping Wang, Ya-Rong Li

**Affiliations:** Department of Integrated Traditional Chinese and Western Medicine, Zhongnan Hospital of Wuhan University, Wuhan University, Wuhan, 430071 China

**Keywords:** Cell proliferation, Complementary therapies, Diabetic nephropathies, Mesangial cells

## Abstract

**Background:**

Dysfunction of glomerular mesangial cells (GMCs) plays an important role in pathogenesis of diabetic nephropathy. Here, we investigated the effects of Dangguibuxue decoction (DBD), an herbal traditional Chinese medicinal (TCM) formula composed of *Astragali Radix* and *Angelicae Sinensis Radix*, on GMC proliferation and fibrogenesis under high-glucose (HG) conditions.

**Methods:**

Sixty male Sprague Dawley rats were divided into 5 groups and administered intragastric 0.9% saline, low concentration DBD (DBD-L, 1.75 g/kg/d), middle concentration DBD (DBD-M, 3.5 g/kg/d), high concentration DBD (DBD-H, 7.0 g/kg/d) and gliclazide (GL, 2 mg/kg/d), respectively, for 1 week, and then their sera were obtained. Rat mesangial cells (HBZY-1 cells) were treated with these sera under HG condition (30 mmol/L).

**Results:**

The proliferation of GMCs under HG conditions was significantly greater than that under normal glucose condition. Low concentration DBD (DBD-L) inhibited proliferation of GMCs after 72-h incubation (*P* < 0.01), while high concentration DBD (DBD-H) inhibited GMCs proliferation at 24, 48 and 72 time points (*P* < 0.01). There was no significant difference between the inhibitory effect of DBD-H and GL sera on GMC proliferation (*P* > 0.05). Furthermore, all concentrations of DBD (DBD-L, DBD-M and DBD-H) significantly decreased the protein expression of ***α***-SMA(***α***-smooth muscle actin) (*P* < 0.01), an indicator of interstitial fibrosis of GMCs. Finally, DBD-L, DBD-M, DBD-H sera obviously inhibited the increase of HYP (hydroxyproline)secretion under HG condition (*P* < 0.01).

**Conclusion:**

Our results demonstrate an inhibitory effect of DBD extract on proliferation and fibrogenesis of GMCs under HG conditions. The potential role of DBD in the treatment of diabetic neuropathy merits further investigation.

## Background

Diabetic nephropathy is the most common cause of end stage renal disease [1]. The condition is associated with typical renal morphological/ultrastructural changes, such as collapse of the charge barrier at the glomerular basement membrane (GBM), excessive amassing and expansion of extracellular matrix (ECM) components, and advanced interstitial fibrosis [2]. Although the precise pathogenetic mechanism of diabetic nephropathy is not fully elucidated, dysfunction of glomerular mesangial cells (GMCs) is thought to play an important role in its pathogenesis.

The dysfunction of GMCs in diabetic nephropathy is attributed to hyperglycemia-induced reprogramming of intracellular metabolism, changes in signaling pathway networks, excessive inflammation and production of reactive oxygen species (ROS) [3, 4].

Dangguibuxue decoction (DBD) is a herbal traditional Chinese medicinal (TCM) formulation that is composed of two herbs, *Astragali Radix* and *Angelicae Sinensis Radix*. Li Dongyuan described DBD for the first time in the Neiwaishang Bianhuo Lun in 1247 AD [5]. DBD has been used as a remedy for various diseases since more than 800 years in China [5]. DBD was shown to enhance bone regeneration [6], attenuate pulmonary fibrosis [7], stimulate proliferation of T-lymphocytes proliferation [8], and treat ailments by phosphorylating estrogen receptors [9]. Therapeutic effects of DBD in kidney-related disorders such as renal anemia and diabetic nephropathy have been recently reported [10–15]. DBD was shown to induce erythropoietin expression via hypoxia-inducible factor-1α signaling pathway in the kidneys [16], to alleviate renal damage in streptozotocin-induced diabetic rats [10], and to reduce adriamycin-induced nephropathy in rats [17]. However, the pharmacological actions of DBD, and the underlying molecular mechanisms in the treatment of diabetic nephropathy, are largely unknown.

The present study is based on hypothesis that DBD may directly affect proliferation of GMCs and renal fibrogenesis, and thus treat diabetic nephropathy. The objective was to determine the effect of DBD on high glucose-induced proliferation and fibrosis in cultured GMCs.

## Methods

### Animals

Sixty male Sprague Dawley rats (~250 g) were purchased from the Wuhan University Animal Center (Wuhan, China) and maintained in specific pathogen-free conditions for 1 week prior to the experiment. All experimental procedures conformed to the NIH guidelines and were approved by the Ethical Review Board and Institutional Animal Care of Zhongnan Hospital of Wuhan University.

### Cell culture

Rat mesangial cells line (HBZY-1 cell) was obtained from American Type Culture Collection (ATCC; Manassas, VA). HBZY-1 cells were cultured in RPMI-1640 medium (purchased from Hyclone, Logan, UT), supplemented with 10% fetal bovine serum (FBS, purchased from Gibco, Grand Island, NY), in a humidified chamber containing 5% CO_2_ at 37 °C as described previously [18].

### Preparation of DBD

The decoction components (*Astragali Radix* and *Angelicae Sinensis Radix*) were prepared by Chinese pharmacy at the Zhongnan Hospital of Wuhan University. The decoction components were soaked in water for 30 min and then decocted to an extract solution (2 mg/mL). High-performance liquid chromatography fingerprinting was used to control the quality of the decoction.

### Preparation of sera containing DBD

The rats were randomly divided into five groups (*n* = 12 per group) and administered intragastric 0.9% saline (control), low concentration DBD (DBD-L, 1.75 g/kg/d), medium concentration DBD (DBD-M, 3.5 g/kg/d), high concentration DBD (DBD-H, 7.0 g/kg/d) and gliclazide (GL, 2 mg/kg/d, purchased from Servier Co. Ltd., France), respectively, for 1 week. At 1 week post-administration, rats were anesthetized using pentobarbital sodium (40 mg/kg), and blood samples collected from abdominal aorta 2 h after the last intragastric administration. Blood samples were stored at 4 °C for 4 h, followed by their centrifugation at 3000 rpm for 30 min to obtain sera.

Sera from the same group were mixed and inactivated by heating in a 56 °C water bath for 30 min. The sera were then filtered using 0.22 μm membrane filters and stored at −80 °C. Sera obtained from the five groups were labeled as control serum, DBD-L serum, DBD-M serum, DBD-H serum and GL serum, respectively.

### Drug administration in cells

HBZY-1 cells were starved in low-serum RPMI-1640 medium (1% FBS) for 24 h. Subsequently, cells were divided into six groups: [1] 10% FBS plus 10% normal serum in 5.6 mmol/L glucose (referred to as NC group); [2] 10% FBS plus 10% normal serum in 30 mmol/L glucose (HG group); [3] 10% FBS plus 10% DBD-L serum in 30 mmol/L glucose (HG + DBD-L group); [4] 10% FBS plus 10% DBD-M serum in 30 mmol/L glucose (HG + DBD-M group); [5] 10% FBS plus 10% DBD-H serum in 30 mmol/L glucose (HG + DBD-H group); and [6] 10% FBS plus 10% GL serum in 30 mmol/L glucose (HG + GL group).

### Evaluation of cell proliferation

Cell proliferation was evaluated by MTT (3-[4,5-dimethylthiazol-2-yl]-2, 5-diphenyltetrazolium bromide) assay as described elsewhere [18, 19]. Briefly, mesangial cells (4 × 10^4^) were plated in 96-well culture plate and were allowed to adhere and spread for 24 h with 100 ul of RPMI 1640 culture medium containing 1% fetal bovine serum. Subsequently, cells were divided into six groups (NC, HG, HG + DBD-L, HG + DBD-M, HG + DBD-H and HG + GL) and treated as mentioned above for 24, 48 and 72 h. At these time-points, 100 μL of MTT solution (5 mg/mL in PBS) was added to the culture well and incubated again for 4 h. The formazan crystals formed were solubilized in 100 μL of dimethyl sulphoxide. The optical density of purple color was measured at 570 nm in a microplate reader. MTT was purchased from Sigma-Aldrich Co. LLC. (San Louis, MO).

### Measurement of Hydroxyproline

Hydroxyproline (HYP) secreted by HBZY-1 cells into the culture medium was measured using a commercial enzyme-linked immunosorbent assay kit (#A030–1, NanJingJianCheng Bioengineering Institute, Nanjing, China). The cells were seeded on to a 48-well plate and allowed to adhere overnight. Cells were starved for 24 h in low-serum RPMI-1640 medium (1% FBS) to synchronize the cell population. The cells were then treated with the extracted sera for 72 h, as described above. The cultured medium was collected and centrifuged at 2000 rpm for 10 min. After discarding the debris, the supernatant was collected for measurement, using ELISA kit, according to the manufacturer’s instructions.

### Western blotting

Cells were washed with cold phosphate-buffered saline (PBS) for three times, lysed in radioimmuneprecipitation assay (RIPA) buffer with protease inhibitor cocktail (purchased from Pierce, Rockford, IL), and used for Western blotting as described [20, 21]. About 30 μg samples were run on 10% SDS-PAGE. The proteins were electro-transferred to nitrocellulose membranes for 3 h. The nitrocellulose membranes were blocked in 5% milk for 1 h and then incubated overnight with rabbit anti-rat monoclonal antibody of α-smooth muscle actin (α-SMA, #ab32575, Abcam, Cambridge, MA) at 4 °C. Then, the membranes were washed by tris-buffered saline with 1% tween 20 (TBST) for three times, and then incubated with horseradish peroxidase-conjugated secondary antibody against rabbit (KPL Gaithersbug, MD).

The membranes were washed three times and the immunoreactive proteins were detected by enhanced chemiluminescence (ECL kit, purchased from GE Healthcare LifeSciences, Piscataway, NJ) on radiographic films (purchased from Kodak, Rochester, NY). The films were scanned and the optical density of blots analyzed using Image J software (NIH). Glyceraldehyde-3-phosphate dehydrogenase (GAPDH) was detected with rabbit anti-rat polyclonal antibody (#ab37168, Abcam) for internal loading control. Each experiment was repeated a minimum of three times.

### Statistical analysis

Data were analyzed with GraphPad Prism-5 statistic software (La Jolla, CA). All values are presented as mean ± Standard error of the mean (SEM). Between-group differences were assessed by Student’s *t*-test or Analysis of Variance (ANOVA) followed by Tukey post-hoc test. *P < 0.05* was considered as statistically significant.

## Results

### DBD inhibits HG-induced proliferation of GMCs

**Fig. 1:**
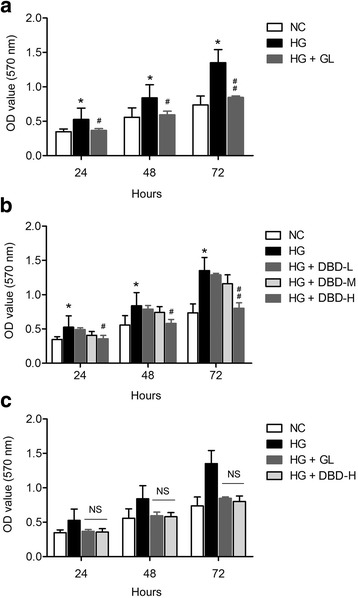
Effects of DBD on proliferation of GMCs under high-glucose conditions (**a**) Proliferation of GMCs under high-glucose (HG) and HG plus gliclazide serum conditions at three time-points (24, 48 and 72 h); **b** Effect of DBD serum on proliferation of GMCs under HG condition at the three time-points; **c** Comparison of the inhibitory action of DBD serum and GL serum on proliferation of GMCs under HG conditions. *P < 0.05 vs. NC (normal control); ^#^ *P* < 0.05 vs. HG. *N* = 6 per group. *DBD, Dangguibuxue decoction; GMCs,* *glomerular* *mesangial cells; GL, gliclazide*

We determined the effects of DBD on GMCs proliferation at 24, 48 and 72 h. The proliferation of GMCs under HG conditions (HG group) was significantly greater than that under normal glucose condition (NC group), at all the three time-points (Fig. 1a). DBD-L serum did not affect the proliferation of GMCs (Fig. 1b). Although DBD-M serum failed to inhibit GMCs proliferation at the 24- and 48-h time-points, it significantly suppressed GMCs proliferation at 72 h (Fig. 1b). DBD-H serum had a remarkable inhibitory effect on proliferation of GMCs at all three time-points (Fig. 1b). Moreover, there was no significant difference in the inhibitory effect of DBD-H serum and GL serum on the proliferation of GMCs (Fig. 1c).

### DBD decreases HG-induced α-SMA expression in GMCs

**Fig. 2:**
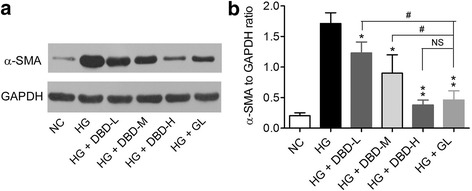
Effects of DBD on α-SMA expression in GMCs. **a** Representative Western blot images showing α-SMA protein expression in cultured GMCs. GAPDH was used as a loading control; **b** Quantitative analysis of α-SMA protein expression in cultured GMCs. *P < 0.05, **P < 0.01 vs. HG; ^#^ *P* < 0.05 vs. HG + GL. *N* = 4 per group. *DBD, Dangguibuxue decoction; α-SMA,α-smooth muscle actin; GMCs,* *glomerular* *mesangial cells; GL, gliclazide; NS, not significant*

Upregulation of ***α***-SMA is an indicator of interstitial fibrosis of GMCs, which has been shown to strongly correlate with the progression of diabetic nephropathy [22]. We found that the ***α***-SMA protein expression in HG-treated GMCs was significantly higher (~8-folds) than that in normal GMCs (Fig. 2a-b). All the three types of extracted sera (DBD-L, DBD-M and DBD-H) decreased the protein expression of ***α***-SMA (Fig. 2a-b). GL also reduced ***α***-SMA protein expression (Fig. 2a-b). There was no significant difference between DBD-H serum and GL serum with respect to their inhibitory effect on ***α***-SMA protein expression (Fig. 2a-b).

### DBD depresses HG-induced HYP secretion in GMCs

**Fig. 3:**
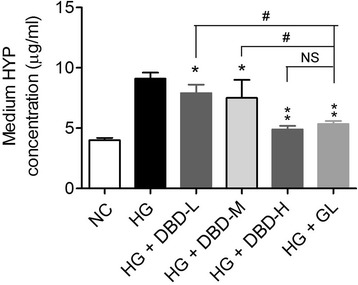
Effects of DBD on HYP secretion in GMCs. Quantitative analysis of HYP secretion in culture medium of GMCs. *P < 0.05, **P < 0.01 vs. HG; ^#^ *P* < 0.05 vs. HG + GL. *N* = 4 per group. *DBD, Dangguibuxue decoction; HYP,* hydroxyproline; *GMCs,* *glomerular* *mesangial cells; GL, gliclazide;* NS, not significant

HYP is another sensitive marker of renal fibrogenesis [23]. HG significantly enhanced HYP secretion from GMCs (Fig. 3). DBD-L, DBD-M, DBD-H and GL sera inhibited the effect of HG on HYP in GMCs (Fig. 3). This inhibitory action was most evident with DBD-H and GL sera (Fig. 3). In addition, there was no difference between DBD-H and GL sera in this respect (Fig. 3).

## Discussion

DBD has been used as a clinical agent against renal dysfunction for more than 1, 000 year in China [10–15]. In recent years, several investigations have highlighted its beneficial action on diabetic nephropathy. For example, Zhang et al. reported that DBD treatment attenuated the increases in fasting blood glucose, lipid, renal kidney/body weight (K/B) ratio, urinary albumin excretion, and creatinine clearance rate in STZ-induced diabetic rats compared with benazepril [10]. In this study, we demonstrated that DBD inhibits proliferation of GMCs, suppresses ***α***-SMA expression and reduces HYP secretion in cultured GMCs under high glucose conditions. These in vitro results reveal the inhibitory effects of DBD on the proliferation and fibrogenesis in GMCs, and suggest a potential role of DBD as a complementary therapeutic strategy for diabetic nephropathy.

The proliferation of GMCs is recognized as a major pathogenic event in the progression of diabetic nephropathy. Several studies have shown that the phenotype of cultured GMCs caused by HG environment mimics the pathophysiological changes in diabetic nephropathy [24]. Gene variations also critically contribute to the renal dysfunction in diabetes [25, 26]. We found that high concentration DBD extract significantly inhibited GMCs proliferation at all three time-points (24, 48 and 72 h), while medium concentration DBD extract only inhibited GMCs proliferation at 72 h. Low concentration DBD extract did not seem to inhibit proliferation of GMCs.

These results indicate that the inhibitory effect of DBD on proliferation of GMCs is dose- and time-dependent. Gao et al. reported that DBD extract reversed high glucose-induced inhibition of endothelial cell migration and proliferation in vitro [27]. Ke et al. also showed that DBD inhibited HG-induced GMCs proliferation and reduced the expression of laminin and type IV collagen in GMCs [28]. Interestingly, DBD was previously reported to enhance proliferation of osteoblasts [29], T-lymphocytes [30], and bone marrow cells [31]. The discrepancy between the effects of DBD on cell proliferation may be due to the differences in culture conditions and cell types.

Fibrogenesis is another critical event in diabetic nephropathy [2, 3, 32]. At least four distinct cell types, including GMCs, bone marrow-derived progenitors, interstitial fibroblasts, and tubular epithelial cells, have been shown to participate in the metaplastic changes in diabetic kidney [33]. GMCs are a specialized type of vascular smooth muscle cells that constitute about 30–40% of the total glomerular cell population [34]. These provide structural support for glomerular capillaries and take part in the regulation of the glomerular filtration rate [34]. In normal conditions, GMCs do not express fibroblast-specific protein [33]. However, the behavior of GMCs changes under diabetic conditions. Hypertrophy of GMCs and mesangial matrix expansion are well-recognized characteristics in diabetic nephropathy [35]. These pathogenetic changes eventually lead to renal fibrosis, GBM thickening and ultimately obliteration of glomerular capillaries [35].

In the present study, DBD significantly attenuated the upregulation of α-SMA in cultured GMCs by HG. α-SMA is absent in normal mesangial matrix; and the upregulation of α-SMA is a marker of myofibroblast fibrogenesis [35]. Moreover, α-SMA also functions as a mechano transducer that responds to signals received at focal adhesions [35]. Thus, the inhibitory action of DBD on α-SMA suggests its potential anti-fibrotic effect that may protect against diabetic nephropathy. HYP is a specific amino acid of collagen; and widely used as a marker for collagen production [36]. We also detected reduced HYP secretion in DBD-treated GMCs, which further supports the anti-fibrotic effect of DBD in diabetic nephropathy.

There are few limitations in our study. The inhibitory action of DBD on GMCs proliferation involves various signaling pathway activation [37–39]. For example, Park et al. reported that HG induces proliferation of GMCs via activation of intercellular adhesion molecule-1 (ICAM-1) [37] Wolf et al. demonstrated that p27 activation is required for the HG-induced proliferation of GMCs [38]. Danesh et al. showed that HG-induced Rho GTPase/p21 signaling contributes to the proliferation of GMCs [39]. We did not explore the underlying molecular mechanisms of the inhibitory action of DBD on the proliferation of GMCs and fibrogenesis. These interesting questions are worth pursuing in future investigations.

## Conclusion

In summary, we demonstrated the inhibitory effect of DBD extract on proliferation and fibrogenesis of rat GMCs under high glucose conditions. The potential role of DBD in treatment and prevention of diabetic neuropathy merits further investigations.
